# Measuring Diet Intake and Gastrointestinal Symptoms in Irritable Bowel Syndrome: Validation of the Food and Symptom Times Diary

**DOI:** 10.14309/ctg.0000000000000103

**Published:** 2019-12-02

**Authors:** Morag Wright-McNaughton, Sebastiaan ten Bokkel Huinink, Christopher M.A. Frampton, Andrew M. McCombie, Nicholas J. Talley, Paula M.L. Skidmore, Richard B. Gearry

**Affiliations:** 1 Department of Human Nutrition, University of Otago, Dunedin, New Zealand;; 2 Department of Medicine, University of Otago, Christchurch, New Zealand;; 3 Department of Medicine, VU Medical Centre, Amsterdam, the Netherlands;; 4 Faculty of Health and Medicine, University of Newcastle, Newcastle, New South Wales, Australia.

## Abstract

**INTRODUCTION:**

Patients with irritable bowel syndrome (IBS) identify food as a trigger for the onset or worsening of gastrointestinal symptoms. Despite this, there is no published validated contemporaneous food and symptom diary to investigate the association between diet and IBS symptoms. The objective of this prospective observational study was to assess the construct validity of a novel food diary and symptom questionnaire, the Food and Symptom Times (FAST) diary, and the predictive validity of the food diary component with relation to fiber and fermentable oligosaccharides, disaccharides, monosaccharides, and polyols consumption and subsequent gastrointestinal symptoms.

**METHODS:**

Fifty-one participants with IBS completed the FAST diary and several legacy instruments. The relationship between the FAST gastroenterological symptoms and legacy instruments was examined using Spearman correlation coefficients. Further statistical analysis investigated the relationship between diet and postprandial gastrointestinal symptoms.

**RESULTS:**

Consistent with *a priori* predictions, the FAST symptoms showed moderate correlations with the most similar Patient-Reported Outcome Measurement Information System gastrointestinal scales (0.328–0.483, *P* < 0.05) and the most similar Gastrointestinal Symptom Rating Scale questions (0.303–0.453, *P* < 0.05), with the exception of the weakly correlated subscale constipation for both instruments (−0.050 to −0.119, *P* > 0.05). The IBS-Quality of Life instrument showed moderate correlations with the FAST symptom abdominal swelling/distension (0.313–0.416, *P* < 0.05). The consumption of a high fermentable oligosaccharides, disaccharides, monosaccharides, and polyols meal was associated with participants with IBS-D experiencing abdominal bloating and participants with IBS-C not experiencing abdominal swelling (*P* < 0.05). The consumption of fiber was correlated with abdominal fullness and bloating in participants with IBS-C (*P* < 0.05).

**DISCUSSION:**

The FAST diary validly measures gastrointestinal symptoms as they occur in people with IBS and correlates these symptoms with specific aspects of diet.

## INTRODUCTION

Irritable bowel syndrome (IBS) is a highly prevalent, chronic functional gastrointestinal disorder (1). There are no validated biomarkers for IBS; thus, diagnosis is based on clinical symptoms of abdominal pain, altered bowel habits, and often abdominal bloating and distension (2). IBS is classified as IBS-D (IBS with diarrhea), IBS-C (IBS with constipation), IBS-M (IBS with mixed bowel habits), or IBS-U (IBS unclassified) based on predominant bowel patterns (2). Patients with IBS frequently attribute foods as triggers for the onset and worsening of their gastrointestinal symptoms (3–6). This postprandial worsening of symptoms typically occurs within 3 hours of eating (7). Fiber, caffeine, spicy foods, alcohol, and fatty food items have been reported as triggers for symptoms, in part, because of their ability to affect gastrointestinal motility (7–17). There is limited and conflicting evidence to support these observed and patient-perceived food-related gastrointestinal symptoms (6).

Conversely, modifying the dietary intake of fermentable oligosaccharides, disaccharides, monosaccharides, and polyols (FODMAPs) has been investigated primarily for treating gastrointestinal symptoms and has shown promising results (18–22). However, despite the published associations between diet and gastrointestinal symptoms in IBS (3,4), there are no validated instruments to evaluate these associations. Furthermore, detailed studies investigating the relationship between food, nutrients, and symptoms in IBS are surprisingly few, in part, because of the controversial role of diet in managing IBS symptoms. The reliance on retrospective questionnaires (23–25) to assess gastrointestinal symptoms is limited because of symptom fluctuation and recall bias; overreporting using these questionnaires is common (26). To concurrently record food intake and gastrointestinal symptoms, we incorporated a real-time gastrointestinal symptom scale into a multiple-day food diary, the Food and Symptom Times (FAST) diary, which could be used for studying patients with IBS.

The primary objective of this prospective observational study was to measure the construct validity of the FAST diary by comparing FAST symptoms with legacy instruments. It was hypothesized there will be modest correlations between the gastrointestinal symptoms and the legacy instruments. A second objective, for which the study was not specifically powered, was to evaluate the associations between fiber and FODMAP consumption and postprandial symptoms.

## METHODS

### Participants

A sample of 51 participants were recruited in New Zealand. Participants aged between 18 and 65 years who satisfied the Rome IV criteria for the diagnosis of IBS (2), which also classifies IBS subtypes, were considered for inclusion in the study. Those with organic gastrointestinal disease or a significant active comorbidity, including cancer, cardiovascular disease, or diabetes, were excluded. A study size of 50 participants was considered reasonable because there are no published data concerning the validation of a time-specific food and symptom diary in IBS.

### Study design

This study was a prospective observational study. All participants gave written informed consent, completed a demographics form, and were assessed using the Rome IV diagnostic criteria for IBS. Eligible participants were given 4 questionnaires (1 collecting feedback about the diary and 3 legacy instruments: the Gastrointestinal Symptom Rating Scale [GSRS], the Irritable Bowel Syndrome Quality of Life [IBS-QOL], and the Patient-Reported Outcome Measurement Information System [PROMIS] gastrointestinal scales) and the FAST diary to complete. Participants were asked to complete the FAST diary on 3 nonconsecutive days including 1 weekend day of a typical week and then to answer the questionnaires. Ethical approval was obtained from the University of Otago Human Ethics Committee (Health) (Reference H16/094).

### FAST diary

The FAST diary was informally pretested by a number of patients with IBS and research staff with extensive experience in the development of similar methodology who provided iterative feedback on the questionnaire and diary. The food diary component of the FAST diary is a standard format used extensively in research (27–30). Real-time 24-hour gastrointestinal symptom scales were embedded within the FAST diary. The FAST diary symptoms are abdominal pain, abdominal swelling/distension, abdominal fullness, and abdominal bloating. Participants recorded the time, duration, and severity of each symptom experienced. Symptom severity ranged from “not bad at all” to “very bad.” Participants also recorded the following information for each bowel motion: Bristol stool type, if there was any straining associated with passage of the bowel motion, if abdominal pain was felt before the bowel motion, how much urgency was experienced, and whether the abdominal pain was relieved or worsened after the bowel motion using a 5-point Likert scale. The overall severity of bowel motions was calculated as a mean of total straining, abdominal pain, and urgency of each bowel motion. See Figure 1, Supplementary Digital Content 1, http://links.lww.com/CTG/A127, for the FAST diary.

### Legacy instruments

Participants completed a range of validated retrospective legacy instruments: the GSRS, the PROMIS gastrointestinal scales, and the IBS-QOL questionnaire. The GSRS is designed to evaluate physical gastrointestinal symptoms during the past week (23). The PROMIS gastrointestinal scales are designed to evaluate patient-reported physical, mental, and social health of patients with gastrointestinal disorders over the past 7 days (24). The IBS-QOL is designed to evaluate health-related quality of life of patients with IBS over the past month (25). Each of these validated questionnaires contains questions that relate directly to questions in the FAST questionnaire. These were used to assess the relationship between the FAST questionnaire and the legacy instruments.

### Food and nutrient composition

The FAST diary recorded each participant's food and beverage intake over 3 nonconsecutive days, which is sufficient to provide information of an individual's habitual dietary intake (30). Nonconsecutive days are routinely used for diet record studies to improve the generalizability of the food record to habitual diet. This information was entered in the dietary assessment software Kai-culator (version 1.15k) developed in the Department of Human Nutrition at the University of Otago. The Kai-culator food and nutrient composition database contains the NZ Institute of Plant and Food Research FOOD files (2014) (31) and recipes that consider moisture and nutrient changes which occur during cooking. Because Kai-culator does not contain all FODMAP nutrients, meals were individually coded according to the Monash University Low FODMAP Diet Application, which contains information about the FODMAP content of common food items (32). Composite food items that were not recorded in the application, such as some baked goods, sauces, and prepared meals, were allocated according to the item's individual ingredients. The FODMAP content of each meal was coded as high or low based on established FODMAP databases held in the Monash FODMAP database (33). Those meals with ingredients classified as medium (yellow) or high (red) FODMAP content on the Monash University Low FODMAP Diet Application were coded as high FODMAP for purposes of this study. Unfortunately, Kai-culator is not able to divide total fiber into soluble and insoluble fractions, so total fiber was reported and used for analyses.

### Data entry and analysis

The legacy instruments were scored in accordance with the instructions for each questionnaire. Data from the FAST symptoms were mapped over respective 24-hour periods and divided into 30-minute sections, with the presence and severity of each symptom recorded. Postprandial symptoms arising within 3 hours of each meal were recorded. Primary analyses investigated the relationship between the mean and maximum FAST symptoms and the legacy instruments; Spearman correlations and ANOVA tests were used to statistically evaluate the strength of these associations.

A meal was defined as any food items consumed over a 30-minute period, excluding just water consumption. Snacks consumed within 30 minutes of a meal were included as part of the meal. A dietitian and experienced nutritionist checked the accuracy of data entry and allocations into Kai-culator. Secondary analyses investigated the association of mealtime FODMAP and fiber intake with postprandial FAST symptoms being assessed using χ^2^ and ANOVA tests, respectively. Each participants' questionnaires and diaries were screened for missing data, and the participants were asked to provide the missing responses where applicable. The symptom window after meals for associations was chosen to be 3 hours for this study based on results from a previous study of gastrointestinal symptoms in patients with IBS after meals (7).

All data were imported into SPSS version 25 (IBM, Armonk, New York), and data from different sources were combined for analyses. Significance levels for all the tests were set at *P* < 0.05.

### Construct validity

The construct validity of a patient-reported outcome such as the FAST symptoms can be established by measuring its relationship with legacy instruments. Comparing logically related measures, such as the FAST symptom abdominal pain and the GSRS abdominal pain, to see whether they are convergent enables the establishment of construct validity if *a priori* expectations are met. We hypothesized *a priori* that the FAST symptoms would modestly and significantly correlate with logically selected subscales and questions from the legacy instruments. Although there should be some correlation between the legacy instrument symptoms reported and the FAST symptoms because they measure the same construct (gastrointestinal symptoms during the same week), it is anticipated that the correlation between these 2 constructs will be modest because long-term recall can differ significantly from real-time recording of symptoms (34) and symptoms can fluctuate throughout the day. Spearman correlation coefficients between the FAST symptoms and each of the logically related scales and questions from the legacy instruments were calculated to measure the strength of this association.

## RESULTS

Eighty-seven people were screened, but 22 did not meet the Rome IV criteria for IBS. Fourteen participants were lost to follow up, leaving a sample of 51 participants (78.5% of the 65 eligible participants) completing the study. Participants' demographic characteristics are described in Table 1. Most of the participants were women (96.1%), 11.8% identified as Maori, and most of the participants had IBS-D. All 51 participants completed the FAST questionnaire fully based on the completeness of answers and the recorded amount of food consumed. The 14 participants who were lost to follow up did not differ significantly as a group from those who were included regarding mean age, sex, and IBS subtype frequency.

**Table 1.**
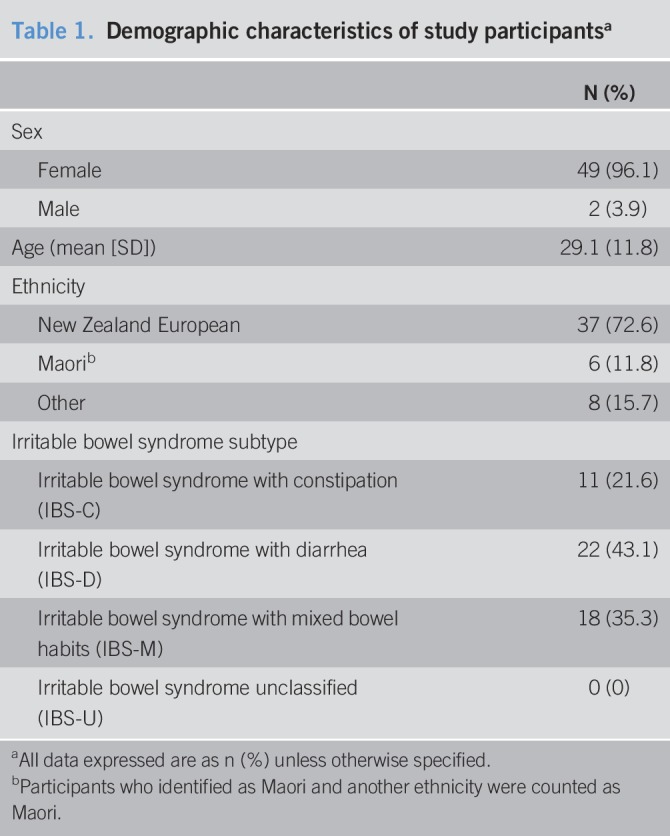
Demographic characteristics of study participants^a^

Participants experienced symptoms after 427 of 782 (54.6%) of all meals consumed. Table 2 describes the proportion of meals for which symptoms arose in the subsequent 3 hours. The most prevalent symptom was abdominal pain; however, when abdominal swelling occurred, this was rated as the most severe symptom experienced (trending toward “quite bad”) and lasted for the longest duration (149 ± 134 minutes). Table 3 documents participant scores for the legacy instruments according to sex and IBS subtype. There were no significant differences between participant scores according to sex. As expected, participants with IBS-C and IBS-D had the highest mean responses for constipation and diarrhea, respectively. Participants with IBS-C reported a higher prevalence of health worry according to the IBS-QOL.

**Table 2.**
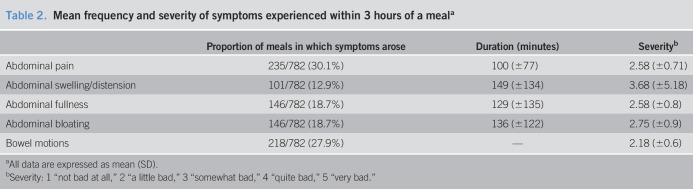
Mean frequency and severity of symptoms experienced within 3 hours of a meal^a^

**Table 3.**
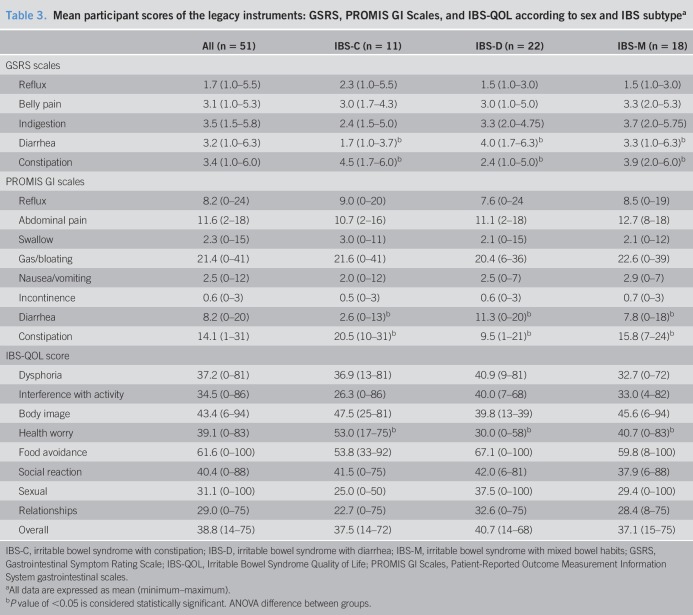
Mean participant scores of the legacy instruments: GSRS, PROMIS GI Scales, and IBS-QOL according to sex and IBS subtype^a^

To determine the construct validity of the FAST diary, correlations between the symptoms and related scales and questions from the legacy instruments were calculated (Table 4). Abdominal fullness was not recorded by the legacy instruments; therefore, correlations were not run with this FAST symptom scale. Legacy instruments' scale of constipation did not significantly correlate with the FAST symptom of bowel motions. The FAST symptoms correlated moderately with logically related PROMIS gastrointestinal scales (0.328–0.483, *P* < 0.05). The FAST symptoms' severity was moderately correlated with logically related GSRS questions (0.303–0.453, *P* < 0.05). Tables 1–4, Supplementary Digital Content 2, http://links.lww.com/CTG/A128, depict mean correlations between the FAST diary and legacy instruments according to IBS subtype.

**Table 4.**
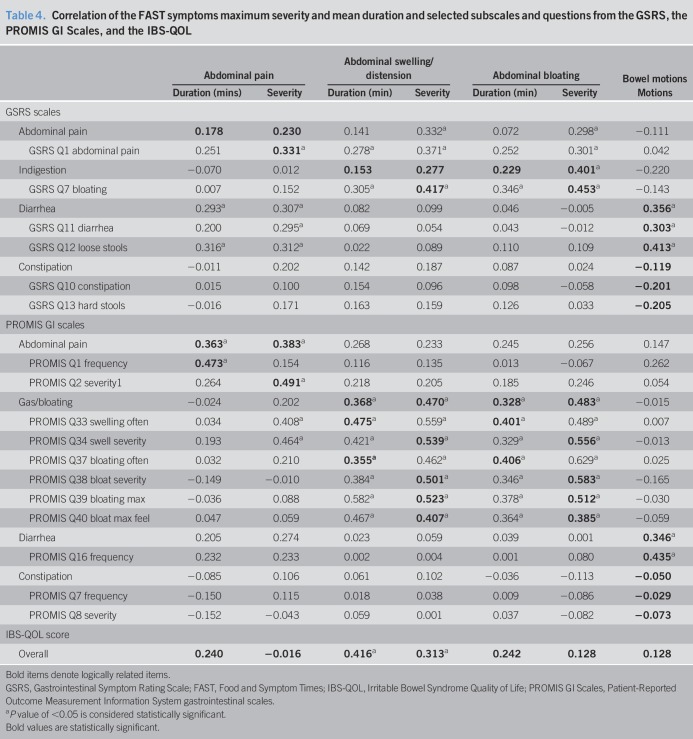
Correlation of the FAST symptoms maximum severity and mean duration and selected subscales and questions from the GSRS, the PROMIS GI Scales, and the IBS-QOL

The participants collectively consumed 782 meals during the study; 607 (77.6%) of these meals contained FODMAPs. A mean of 5.12 (±1.11) meals were consumed daily. For 427 (54.6%) of the meals eaten, participants experienced symptoms within 3 hours; 334 (78.2%) of these meals contained FODMAPs. Table 5 describes the mean severity and duration of symptoms that occurred within 3 hours of a meal containing FODMAPs. The proportion of meals containing FODMAPs was similar for both participants with IBS-D (241/304, 79.3%) and IBS-C (124/166, 74.7%).

**Table 5.**
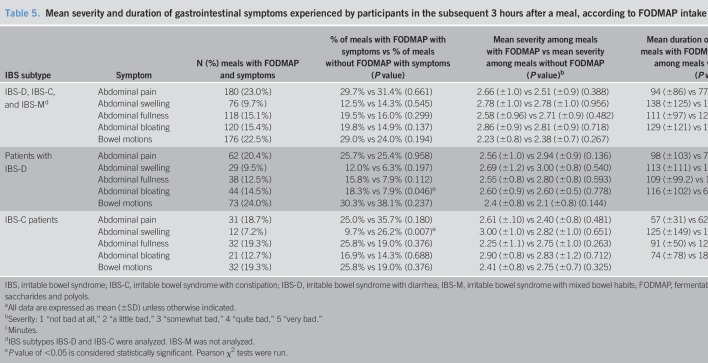
Mean severity and duration of gastrointestinal symptoms experienced by participants in the subsequent 3 hours after a meal, according to FODMAP intake and IBS subtype^a^

No significant associations between the gastrointestinal symptoms in the presence or absence of FODMAP or fiber were seen when the whole sample of participants were analyzed, with the exception of the amount of fiber consumed and the presence of abdominal fullness (*P* < 0.05). Table 6 describes the mean grams of fiber consumed in a meal according to whether symptoms arose in the subsequent 3 hours after consumption. The mean fiber content of all meals was 4.91 g. Participants consumed a mean of 13.94 (SD ± 63.22) grams of fiber daily.

**Table 6.**
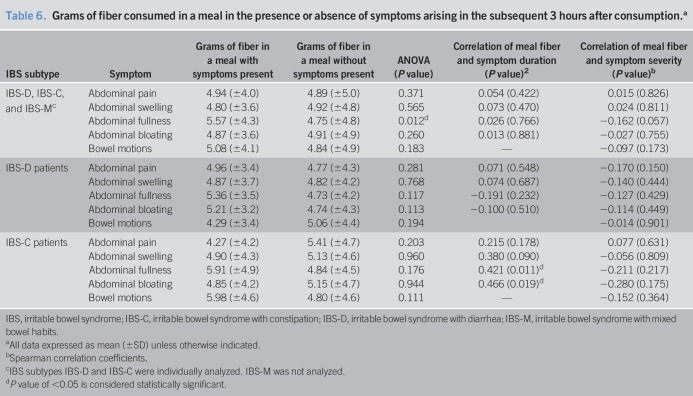
Grams of fiber consumed in a meal in the presence or absence of symptoms arising in the subsequent 3 hours after consumption.^a^

FODMAP intake was associated with abdominal bloating in participants with IBS-D (*P* = 0.046) and with abdominal swelling in participants with IBS-C (*P* = 0.007). For participants with IBS-D, there was a nonsignificant trend for a higher percentage of abdominal swelling, abdominal fullness, and abdominal bloating symptoms after a meal containing FODMAP, compared with a meal not containing FODMAP for which symptoms arose. In participants with IBS-C, moderate correlations between fiber consumption and the duration of postprandial symptoms experienced were found for abdominal fullness (*P* = 0.011) and for abdominal bloating (*P* = 0.019).

## DISCUSSION

We have developed and validated the FAST diary, a novel real-time gastrointestinal symptom scale embedded in a food diary, which records the symptoms experienced by participants at the time of occurrence. To our knowledge, this is the first published study that has used real-time symptom scales to assess gastrointestinal symptoms in adults with IBS and relate these directly to diet. The results show that the FAST symptoms show moderate construct validity when compared with the legacy instruments. Stronger correlations for the severity and for the length of time that the gastrointestinal symptoms were experienced arose when correlated with related legacy instrument questions. Secondary analyses of food intake in relation to gastrointestinal symptoms were underpowered but mostly trended in the anticipated direction; FODMAPs consumed at a meal showed a trend for a greater percentage of symptoms arising after the meal for participants with IBS-D, and fiber intake was correlated with symptom duration in participants with IBS-C.

The most prevalent postprandial symptoms that participants experienced were abdominal pain and bowel motions. Because participants were included in the study based on the presentation of these symptoms according to the Rome IV diagnostic criteria (2), this is not surprising. Abdominal swelling was the least prevalent symptom experienced within 3 hours of a meal; however, when this symptom arose, it was the most severe. In comparison, other symptoms experienced were mild to moderate in severity. The FAST symptom scales are likely to be more accurate in reporting symptom occurrence and severity than retrospective methods because of symptoms being recorded concurrently; this is likely to mitigate recall bias by minimizing reliance on long-term memory (34). Minimizing the participant's recall bias in this way will improve the accuracy of participants reporting of both their gastrointestinal symptoms and food intake. However, although most participants were mostly accurate in reporting their symptoms, entering in symptoms at the end of the day, backfilling may have occurred. The mild severity of these symptoms experienced by the study participants is less severe than retrospectively reported symptoms (35–38). This could reflect a milder phenotype of participants in this study or could be because of the overreporting of symptoms found in retrospective studies compared with real-time or end-of-day measures (26,39). As such, the correlations between the symptoms in the FAST diary and the logically related legacy instruments' scales and questions were weak to moderate as hypothesized. Consistent with this observation, the mild severity of the gastrointestinal symptoms experienced may have led to a floor effect (40), resulting in weak to moderate correlations with the legacy instruments.

In our study, most (83.3%) of the FAST symptoms had significantly moderate correlation coefficients when compared with the logically related legacy instrument questions of the GSRS and the PROMIS gastrointestinal scales. Previous research has found moderate correlation coefficients, classified as (0.30–0.60), to show good construct validity when assessing associations between gastrointestinal symptom scales and questionnaires (24,25,36,41,42). Only legacy questions pertaining to constipation were not significantly correlated with FAST symptoms. Because IBS-C was the least populous IBS subtype in the present study, this may account for the lack of significance when comparing the legacy instrument's constipation scales with the FAST symptom of bowel motions.

Abdominal swelling/distension was the least prevalent symptom reported, perhaps in part because of the similarity and potential difficulty distinguishing this from the symptom of abdominal bloating. Traditionally, these terms were used interchangeably in the literature; bloating is now defined as a subjective feeling of increased abdominal pressure which may or may not be accompanied by the objective increase in abdominal girth defined as distension (43). Chang et al. (44) suggest that although most participants with IBS experience abdominal bloating associated with abdominal swelling/distension, 24% experienced abdominal bloating alone. Similarly, we found that 31% more meals resulted in postprandial abdominal bloating (146 meals) than in abdominal swelling/distension (101 meals). Because of participants' ability to distinguish between the subjective feeling of bloating and objective distension consistent with previous research, we believe both these measures should be included in the FAST diary.

Although not powered to do so, the present study investigated the relationship between FAST symptoms and the consumption of fiber and FODMAPs within that meal. Although fiber has traditionally been effective in treating constipation, studies have found that fiber may worsen gastrointestinal symptoms (17,45–47). Higher fiber intake at a meal was associated with abdominal fullness, with participants with IBS-C experiencing moderate correlations between fiber intake and the duration of abdominal fullness and bloating. Using the FAST diary in conjunction with a diet calculator that could split total fiber into soluble and insoluble would also be ideal for studies in which fiber was a key variable being studied. Low FODMAP diets can alleviate abdominal pain, bloating, and diarrhea (6,20,22,38,48). In our study, independent of the IBS subtype, FODMAP consumption was found not to affect the severity or duration of symptoms. Participants with IBS-D experienced abdominal bloating more frequently after a FODMAP-containing meal (*P* = 0.046). Conversely, for participants with IBS-C, the consumption of FODMAPs at a meal was found to be associated with less occurrence of abdominal swelling (*P* = 0.007).

These pilot data confirm the feasibility of using the FAST diary to examine the relationship between specific ingredients in meals and gastrointestinal symptoms. However, there are a range of variables that should be optimized based on the food component being examined and the putative mechanism of action by which each ingredient causes symptoms. For example, the symptom window could be wider than 3 hours because of variability in gut transit when examining the effect of some ingredients that may cause symptoms over a longer period than some other foods (49–51). This is particularly true of the effects of oligosaccharides and symptom generation through colonic fermentation (49). Regarding FODMAPs, larger studies need to be performed with more participants examining the effects of individual FODMAPs on gut symptoms. FODMAP intake should also be measured quantitatively to understand the nature of any associations between intake and symptoms more thoroughly. Finally, larger cohorts will enable subphenotypes of IBS to be studied individually and in combination to understand where dietary interventions are likely to be most effective.

This study has a number of limitations. The participants were predominantly women, and the FAST diary needs to be evaluated in men. The sample size was small but within acceptable limits for a validation study. Participant numbers were determined in advance and were acceptable because of the specialized population being studied. Future research should investigate the discriminant validity of the FAST diary in healthy controls and other long-term conditions.

The FAST diary has a wide range of potential uses. In clinical practice, this may provide dietitians with a more accurate means of identifying food triggers for people with gastrointestinal symptoms and enable dietitians to more objectively measure the effectiveness of their clinical care. For researchers, the diaries may be useful for identifying food triggers for symptoms across a population or assessing whether diet-based treatments for a range of disorders are associated with gastrointestinal symptoms.

In conclusion, the FAST diary has predictive and construct validity for the evaluation of gastrointestinal symptoms in patients with IBS. To our knowledge, this is the first published instrument to evaluate the relationship between diet and gastrointestinal symptoms. The construct validity process described in this study shows that the FAST symptoms demonstrate moderate correlations with legacy instruments. Although there is evidence that the FAST diary may predict the intake of selected nutrients associated with gastrointestinal symptoms, to ensure the reliability of these associations, future research should investigate trialing the diary in larger populations representing all IBS subtypes and within populations with other lower gastrointestinal condition.

## CONFLICTS OF INTEREST

**Guarantor of the article:** Richard B. Gearry, MBChB, PhD.

**Specific author contributions:** planning of the study: P.M.L.S., R.B.G., and N.J.T. Data collection: M.W.-M. and S.t.B.H. Data analysis and interpretation: M.W.-M., C.M.A.F., and A.M. Drafting the manuscript: M.W.-M. Critical feedback for the manuscript: P.M.L.S., R.B.G., N.J.T., S.t.B.H., M.W.-M., C.M.A.F., and A.M.M. All authors provided critical feedback to the manuscript and approved the final manuscript.

**Financial support:** This study was funded in part by the High Value Nutrition National Science Challenge from the Ministry for Business, Innovation and Employment, New Zealand.

**Potential competing interests:** None to report.

**Study Highlights**

**WHAT IS KNOWN**

✓ Food may trigger gastrointestinal symptoms.
✓ Gastrointestinal symptoms fluctuate throughout the day.

**WHAT IS NEW HERE**

✓ The FAST diary is a novel instrument used to investigate the relationship between gastrointestinal symptoms and diet in IBS.
✓ The FAST diary correlates moderately with other instruments that record gastrointestinal symptoms and IBS quality of life over a longer period of time.

**TRANSLATIONAL IMPACT**

✓ The FAST diary provides a novel instrument for assessing the relationship between diet and gastrointestinal symptoms.

## Supplementary Material

SUPPLEMENTARY MATERIAL
