# Supplementary material for: Measuring Diet Intake and Gastrointestinal Symptoms in Irritable Bowel Syndrome: Validation of the Food and Symptom Times Diary
Source: Clin Transl Gastroenterol. 2019 Dec 2;10(12):e00103. doi: 10.14309/ctg.0000000000000103 (PMC6970560; doi:10.14309/ctg.0000000000000103)
Supplement: SUPPLEMENTARY MATERIAL [file ct9-10-e00103-s001.docx]

**FAST Diary Food and Beverage Intake Day 1 Date:____________**

| **Meal** | **When** | **Where** | **Who with** | **Food or Drink** | **Brand and details** | **Preparation/**  **Cooking** | **Quantity** |
| --- | --- | --- | --- | --- | --- | --- | --- |
|  |  |  |  |  |  |  |  |
|  |  |  |  |  |  |  |  |
|  |  |  |  |  |  |  |  |
|  |  |  |  |  |  |  |  |
|  |  |  |  |  |  |  |  |
|  |  |  |  |  |  |  |  |
|  |  |  |  |  |  |  |  |
|  |  |  |  |  |  |  |  |
|  |  |  |  |  |  |  |  |
|  |  |  |  |  |  |  |  |
|  |  |  |  |  |  |  |  |
|  |  |  |  |  |  |  |  |
|  |  |  |  |  |  |  |  |
|  |  |  |  |  |  |  |  |
|  |  |  |  |  |  |  |  |
|  |  |  |  |  |  |  |  |
|  |  |  |  |  |  |  |  |
|  |  |  |  |  |  |  |  |
|  |  |  |  |  |  |  |  |
|  |  |  |  |  |  |  |  |
|  |  |  |  |  |  |  |  |
|  |  |  |  |  |  |  |  |

**FAST Diary Gastrointestinal Symptoms Day 1 Date: ______________**

**Food intake**


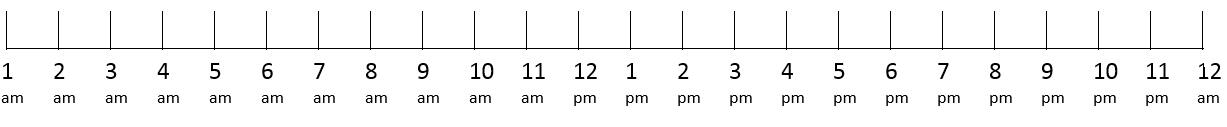


Please mark on the scale if you had any of the symptoms over the last 24 hours, and write the severity of the symptoms using the following scale:

*1 Not bad at all 2 A little bad 3 Somewhat bad 4 Quite bad 5 Very bad*

**Symptom 1. Abdominal pain None ☐**


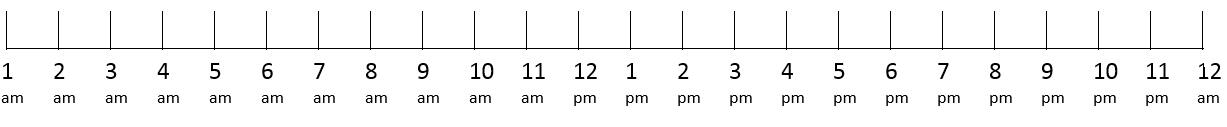


**Symptom 2. Abdominal swelling/distension None ☐**


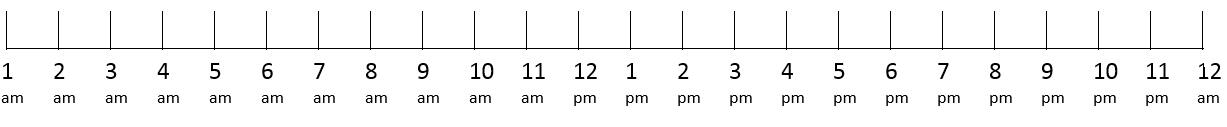


**Symptom 3. Abdominal fullness None ☐**


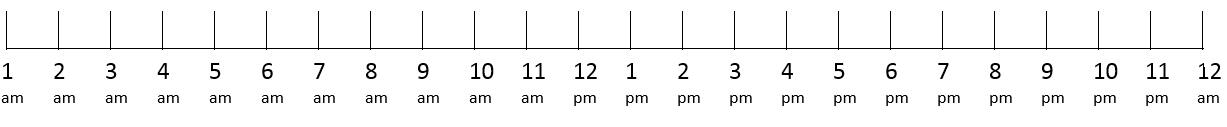


**Symptom 4. Abdominal bloating None ☐**


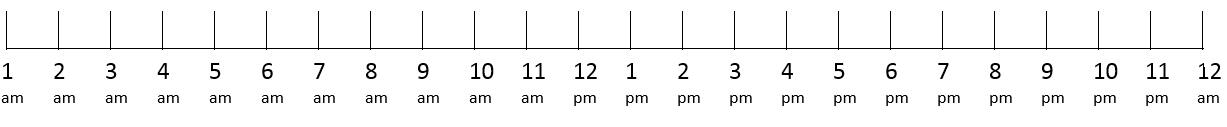


**If you had a Bowel Motion in the last 24 hours please mark on the scale. If you didn’t please tick the box.**

**Bowel motions No bowel motions ☐**


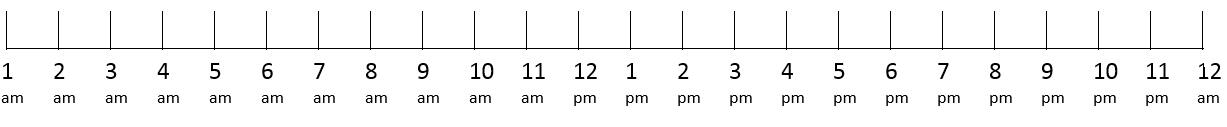


**FAST Diary Bowel Motion Chart Day 1 Date: ______________**

Please complete the following chart reporting symptoms with each bowel motion that you passed in the last 24 hours:


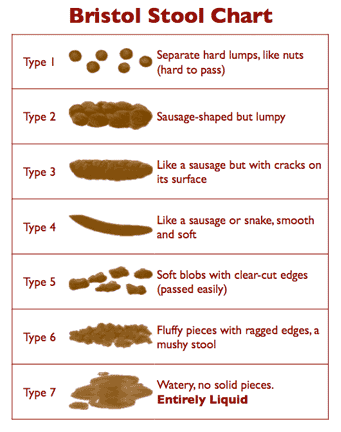


How much did you strain to pass the bowel motion?

1. Not at all
2. Slightly strain
3. Moderately strain
4. Significantly strain
5. Unable to empty bowel

Did you have abdominal pain before your bowel motion?

1. No abdominal pain
2. Not bad at all
3. A little bad
4. Somewhat bad
5. Quite bad
6. Very bad

How much urgency do you experience when you need to have a bowel motion?

1. Not at all
2. A little urgency
3. I have to hurry
4. I have to go immediately
5. I am incontinent (unable to control the urge and had an accident)

|  | **BM1** | **BM2** | **BM3** | **BM4** | **BM5** | **BM6** | **BM7** | **BM8** | **BM9** |
| --- | --- | --- | --- | --- | --- | --- | --- | --- | --- |
| **Bristol stool type**  *(please write the number)* |  |  |  |  |  |  |  |  |  |
| **How much did you strain?**  *(please write the number)* |  |  |  |  |  |  |  |  |  |
| **Abdominal pain before BM?**  *(please write the number)* |  |  |  |  |  |  |  |  |  |
| **Urgency?**  *(please write the number)* |  |  |  |  |  |  |  |  |  |
| **Abdominal pain relieved by BM?**  *(Yes/No/Not Applicable)* |  |  |  |  |  |  |  |  |  |
| **Abdominal pain worsened by BM?**  *(Yes/No/Not Applicable)* |  |  |  |  |  |  |  |  |  |
