# Supplementary material for: Measuring Diet Intake and Gastrointestinal Symptoms in Irritable Bowel Syndrome: Validation of the Food and Symptom Times Diary
Source: Clin Transl Gastroenterol. 2019 Dec 2;10(12):e00103. doi: 10.14309/ctg.0000000000000103 (PMC6970560; doi:10.14309/ctg.0000000000000103)
Supplement: SUPPLEMENTARY MATERIAL [file ct9-10-e00103-s002.docx]

**Supporting Table 1** Correlation of the mean duration and severity of FAST symptoms and selected subscales and questions from the GSRS, the PROMIS, and the IBS-QOL

|  | Abdominal Pain | | Abdominal Swelling/  Distension | | Abdominal Bloating | | Bowel Motions |
| --- | --- | --- | --- | --- | --- | --- | --- |
|  | Duration  (minutes) | Severity | Duration  (minutes) | Severity | Duration  (minutes) | Severity | Motions |
| **GSRS Scales** | | | | | | | |
| Abdominal pain | **0.178** | **0.214** | 0.141 | 0.156 | 0.072 | 0.125 | -0.111 |
| GSRS Q1  Abdo- pain | 0.251 | **0.285*** | 0.278* | 0.271 | 0.252 | 0.267 | 0.042 |
| Indigestion | -0.070 | -0.030 | **0.153** | **0.198** | **0.229** | **0.303*** | -0.220 |
| GSRS Q7  Bloating | 0.007 | 0.054 | 0.305* | **0.342*** | 0.346* | **0.404*** | -0.143 |
| Diarrhoea | **0.293*** | **0.333*** | 0.082 | 0.086 | 0.046 | 0.058 | **0.356*** |
| GSRS Q11  Diarrhoea | 0.200 | 0.223 | 0.069 | 0.066 | 0.043 | 0.055 | **0.303*** |
| GSRS Q12  Loose stools | **0.316*** | **0.377*** | 0.022 | 0.041 | 0.110 | 0.123 | **0.413*** |
| Constipation | -0.011 | 0.071 | 0.142 | 0.127 | 0.087 | 0.121 | **-0.119** |
| GSRS Q10  Constipation | 0.015 | 0.070 | 0.154 | 0.133 | 0.098 | 0.110 | **-0.201** |
| GSRS Q13  Hard stools | -0.016 | 0.060 | 0.163 | 0.129 | 0.126 | 0.150 | **-0.205** |
| **PROMIS GI Scales** | | | | | | | |
| Abdominal pain | **0.363*** | **0.435*** | 0.268 | 0.254 | 0.245 | 0.285* | 0.147 |
| PROMIS Q1  Frequency | **0.473*** | 0.469* | 0.116 | 0.109 | 0.013 | 0.028 | 0.262 |
| PROMIS Q2  Severity | 0.264 | **0.340*** | 0.218 | 0.186 | 0.185 | 0.211 | 0.054 |
| Gas/bloating | -0.024 | 0.040 | **0.368*** | **0.410*** | **0.328*** | **0.380*** | -0.015 |
| PROMIS Q33  Swelling often | 0.034 | 0.113 | **0.475*** | 0.486* | **0.401*** | 0.439* | 0.007 |
| PROMIS Q34  Swell severity | 0.193 | 0.253 | 0.421* | **0.445*** | 0.329* | **0.378*** | -0.013 |
| PROMIS Q37  Bloating often | 0.032 | 0.109 | 0.355* | 0.377* | **0.406*** | 0.471* | 0.025 |
| PROMIS Q38  Bloat severity | -0.149 | -0.144 | 0.384* | **0.402*** | 0.346* | **0.414*** | -0.165 |
| PROMIS Q39  Bloating max | -0.036 | 0.030 | 0.582* | **0.592*** | 0.378* | **0.438*** | -0.030 |
| PROMIS Q40  Bloat max feel | 0.047 | 0.121 | 0.467* | **0.467*** | 0.364* | **0.415*** | -0.059 |
| Diarrhea | 0.205 | 0.232 | 0.023 | 0.029 | 0.039 | 0.044 | **0.346*** |
| PROMIS Q16  Frequency | 0.232 | 0.104 | 0.002 | 0.001 | 0.001 | 0.010 | **0.435*** |
| Constipation | -0.085 | -0.008 | 0.061 | 0.059 | -0.036 | -0.015 | **-0.050** |
| PROMIS Q7  Frequency | -0.150 | -0.102 | 0.018 | 0.026 | 0.009 | 0.022 | **-0.029** |
| PROMIS Q8  Severity | -0.152 | -0.095 | 0.059 | 0.052 | 0.037 | 0.045 | **-0.073** |
| **IBS-QOL Score** | | | | | | | |
| Overall | **0.240** | **0.266** | **0.416*** | **0.414*** | **0.242** | **0.256** | **0.128** |

***** P-value of <0.05 is considered statistically significant.

**Bold** items denote logically related items.

GSRS, gastrointestinal symptom rating scale; IBS-QOL, irritable bowel syndrome quality of life; PROMIS GI, patient reported outcome measurement information system gastrointestinal scales.

**Supporting Table 2** Correlation of the mean FAST symptoms and selected subscales and questions from the GSRS, the PROMIS GI, and the IBS-QOL in IBS-D participants

|  | Abdominal Pain | | Abdominal Swelling/  Distension | | Abdominal Bloating | | Bowel Motions |
| --- | --- | --- | --- | --- | --- | --- | --- |
|  | Duration  (minutes) | Severity | Duration  (minutes) | Severity | Duration  (minutes) | Severity | Motions |
| **GSRS Scales** | | | | | | | |
| Abdominal pain | **-0.085** | **-0.102** | 0.117 | 0.122 | -0.016 | 0.043 | -0.006 |
| GSRS Q1  Abdo- pain | 0.169 | **0.184** | 0.252 | 0.267 | 0.212 | 0.258 | 0.112 |
| Indigestion | -0.247 | -0.274 | **0.009** | **0.018** | **0.051** | **0.133** | -0.383 |
| GSRS Q7  Bloating | 0.037 | 0.047 | 0.427 | **0.422** | 0.393 | **0.457*** | -0.033 |
| Diarrhoea | 0.085 | 0.143 | 0.099 | 0.110 | 0.008 | 0.029 | **0.513*** |
| GSRS Q11  Diarrhoea | 0.103 | 0.152 | 0.037 | 0.025 | -0.137 | -0.135 | **0.465*** |
| GSRS Q12  Loose stools | 0.137 | 0.164 | 0.092 | 0.091 | 0.120 | 0.154 | **0.529*** |
| Constipation | 0.162 | 0.198 | 0.087 | 0.083 | 0.071 | 0.047 | **0.300** |
| GSRS Q10  Constipation | 0.226 | 0.262 | 0.086 | 0.075 | -0.045 | -0.091 | **0.220** |
| GSRS Q13  Hard stools | 0.254 | 0.269 | 0.212 | 0.207 | 0.204 | 0.159 | **0.056** |
| **PROMIS GI Scales** | | | | | | | |
| Abdominal pain | **0.271** | **0.326** | 0.067 | 0.061 | 0.250 | 0.278 | 0.342 |
| PROMIS Q1  Frequency | **0.393** | 0.361 | -0.186 | -0.202 | -0.012 | -0.015 | 0.356 |
| PROMIS Q2  Severity | 0.346 | **0.410** | 0.280 | 0.282 | 0.388 | 0.429* | 0.254 |
| Gas/bloating | -0.066 | 0.004 | **0.521*** | **0.546*** | **0.431*** | **0.478*** | -0.043 |
| PROMIS Q33  Swelling often | 0.064 | 0.142 | **0.540*** | 0.569* | **0.443*** | 0.473* | 0.204 |
| PROMIS Q34  Swell severity | 0.256 | 0.313 | 0.516* | **0.553*** | 0.527* | **0.543*** | 0.047 |
| PROMIS Q37  Bloating often | -0.152 | -0.085 | **0.375** | 0.386 | **0.515*** | 0.549* | -0.008 |
| PROMIS Q38  Bloat severity | -0.256 | -0.209 | 0.460* | **0.484*** | 0.355 | **0.417** | -0.126 |
| PROMIS Q39  Bloating max | -0.047 | -0.002 | 0.577* | **0.599*** | 0.397 | **0.459*** | -0.186 |
| PROMIS Q40  Bloat max feel | -0.018 | 0.018 | 0.567* | **0.569*** | 0.383 | **0.065** | 0.057 |
| Diarrhea | 0.037 | 0.071 | -0.053 | -0.038 | -0.050 | -0.049 | **0.471*** |
| PROMIS Q16  Frequency | 0.131 | 0.136 | -0.090 | -0.099 | -0.126 | -0.098 | **0.410** |
| Constipation | 0.087 | 0.134 | 0.094 | 0.102 | 0.006 | -0.023 | **0.251** |
| PROMIS Q7  Frequency | 0.167 | 0.179 | 0.130 | 0.120 | 0.233 | 0.191 | **0.030** |
| PROMIS Q8  Severity | 0.287 | 0.294 | 0.225 | 0.211 | 0.247 | 0.201 | **0.069** |
| **IBS-QOL Score** | | | | | | | |
| Overall | **0.074** | **0.120** | **0.333** | **0.357** | **-0.013** | **-0.001** | **0.184** |

***** P-value of <0.05 is considered statistically significant.

**Bold** items denote logically related items. GSRS, gastrointestinal symptom rating scale; IBS-QOL, irritable bowel syndrome quality of life; PROMIS GI Scales, patient reported outcome measurement information system gastrointestinal scales; IBS-D, irritable bowel syndrome with diarrhoea.

**Supporting Table 3** Correlation of the mean FAST symptoms and selected subscales and questions from the GSRS, the PROMIS GI, and the IBS-QOL in IBS-C participants

|  | Abdominal Pain | | Abdominal Swelling/  Distension | | Abdominal Bloating | | Bowel Motions |
| --- | --- | --- | --- | --- | --- | --- | --- |
|  | Duration  (minutes) | Severity | Duration  (minutes) | Severity | Duration  (minutes) | Severity | Motions |
| **GSRS Scales** | | | | | | | |
| Abdominal pain | **0.314** | **0.346** | 0.308 | 0.237 | 0.156 | 0.359 | 0.076 |
| GSRS Q1  Abdo- pain | 0.095 | **0.114** | 0.620* | 0.676* | 0.280 | 0.378 | 0.424 |
| Indigestion | -0.071 | -0.041 | **0.532** | **0.722** | **0.519** | **0.671*** | 0.165 |
| GSRS Q7  Bloating | -0.089 | -0.140 | 0.462 | **0.657*** | 0.533 | **0.603** | 0.150 |
| Diarrhoea | 0.333 | 0.449 | 0.271 | 0.125 | -0.032 | -0.057 | **0.282** |
| GSRS Q11  Diarrhoea | 0.000 | 0.224 | 0.491 | 0.488 | 0.076 | 0.075 | **0.539** |
| GSRS Q12  Loose stools | 0.244 | 0.418 | 0.353 | 0.351 | 0.196 | 0.250 | **0.300** |
| Constipation | 0.387 | 0.395 | 0.494 | 0.359 | -0.012 | 0.129 | **0.470** |
| GSRS Q10  Constipation | 0.451 | 0.430 | 0.402 | 0.244 | 0.025 | 0.103 | **0.053** |
| GSRS Q13  Hard stools | -0.153 | -0.130 | 0.460 | 0.365 | 0.213 | 0.296 | **0.182** |
| **PROMIS GI Scales** | | | | | | | |
| Abdominal pain | **0.547** | **0.530** | 0.398 | 0.264 | -0.028 | 0.100 | 0.302 |
| PROMIS Q1  Frequency | **0.605*** | 0.637* | 0.002 | -0.070 | -3.29 | -0.150 | 0.324 |
| PROMIS Q2  Severity | -0.005 | **0.114** | 0.305 | 0.183 | 0.051 | 0.065 | -0.036 |
| Gas/bloating | 0.082 | -0.009 | **0.376** | **0.553** | **0.455** | **0.551** | -0.024 |
| PROMIS Q33  Swelling often | -0.544 | -0.450 | **0.762*** | 0.791* | **0.635** | 0.596 | 0.404 |
| PROMIS Q34  Swell severity | 0.388 | 0.380 | 0.507 | **0.547** | 0.039 | **0.209** | 0.371 |
| PROMIS Q37  Bloating often | 0.559 | 0.592 | **0.248** | 0.246 | **-0.036** | 0.244 | 0.182 |
| PROMIS Q38  Bloat severity | -0.053 | -0.224 | 0.637* | **0.657*** | 0.553 | **0.612** | 0.155 |
| PROMIS Q39  Bloating max | -0.089 | -0.127 | 0.793* | **0.757*** | 0.744* | **0.769*** | 0.236 |
| PROMIS Q40  Bloat max feel | 0.195 | 0.156 | 0.575 | **0.511** | 0.502 | **0.623** | -0.122 |
| Diarrhea | 0.077 | 0.183 | 0.181 | 0.238 | 0.118 | 0.135 | **0.169** |
| PROMIS Q16  Frequency | 0.070 | 0.244 | 0.353 | 0.438 | -0.071 | 0.017 | **0.690*** |
| Constipation | 0.114 | 0.196 | 0.462 | 0.259 | -0.021 | -0.064 | **0.468** |
| PROMIS Q7  Frequency | -0.155 | 0.087 | 0.372 | 0.421 | -0.151 | -0.077 | **0.659*** |
| PROMIS Q8  Severity | -0.029 | -0.042 | 0.519 | 0.308 | 0.195 | 0.243 | **0.025** |
| **IBS-QOL Score** | | | | | | | |
| Overall | **0.365** | **0.345** | **0.652*** | **0.463** | **0.325** | **0.382** | **0.043** |

***** P-value of <0.05 is considered statistically significant.

**Bold** items denote logically related items. GSRS, gastrointestinal symptom rating scale; IBS-QOL, irritable bowel syndrome quality of life; PROMIS GI Scales, patient reported outcome measurement information system gastrointestinal scales; IBS-C, irritable bowel syndrome with constipation.

|  | Abdominal Pain | | Abdominal Swelling/  Distension | | Abdominal Bloating | | Bowel Motions |
| --- | --- | --- | --- | --- | --- | --- | --- |
|  | Duration  (minutes) | Severity | Duration  (minutes) | Severity | Duration  (minutes) | Severity | Motions |
| **GSRS Scales** | | | | | | | |
| Abdominal pain | **0.320** | **0.418** | 0.128 | 0.165 | 0.203 | 0.255 | -0.156 |
| GSRS Q1  Abdo- pain | 0.487* | **0.554*** | 0.080 | 0.100 | 0.260 | 0.292 | -0.139 |
| Indigestion | 0.186 | 0.268 | **0.088** | **0.128** | **0.312** | **0.360** | -0.357 |
| GSRS Q7  Bloating | 0.055 | 0.190 | -0.022 | **0.018** | 0.101 | **0.192** | -0.363 |
| Diarrhoea | 0.224 | 0.309 | 0.160 | 0.210 | 0.245 | 0.359 | **-0.041** |
| GSRS Q11  Diarrhoea | 0.162 | 0.179 | 0.145 | 0.167 | 0.310 | 0.402 | **-0.110** |
| GSRS Q12  Loose stools | 0.214 | 0.299 | -0.050 | 0.007 | 0.233 | 0.302 | **0.032** |
| Constipation | -0.086 | 0.049 | -0.266 | -0.233 | -0.224 | -0.156 | **-0.446** |
| GSRS Q10  Constipation | 0.042 | 0.103 | -0.008 | 0.031 | 0.001 | 0.010 | **-0.336** |
| GSRS Q13  Hard stools | -0.018 | 0.113 | -0.246 | -0.242 | -0.249 | -0.153 | **-0.509*** |
| **PROMIS GI Scales** | | | | | | | |
| Abdominal pain | **0.271** | **0.376** | 0.317 | 0.340 | 0.326 | 0.442 | -0.308 |
| PROMIS Q1  Frequency | **0.219** | 0.213 | 0.638* | 0.661* | 0.176 | 0.257 | -0.063 |
| PROMIS Q2  Severity | 0.218 | **0.308** | 0.049 | 0.073 | 0.009 | 0.099 | -0.314 |
| Gas/bloating | 0.094 | 0.212 | **0.088** | **0.157** | **0.130** | **0.247** | -0.122 |
| PROMIS Q33  Swelling often | 0.397 | 0.471 | **0.169** | 0.198 | **0.219** | 0.261 | -0.362 |
| PROMIS Q34  Swell severity | 0.267 | 0.344 | 0.266 | **0.273** | 0.112 | **0.177** | -0.216 |
| PROMIS Q37  Bloating often | 0.156 | 0.248 | **0.371** | 0.381 | **0.352** | 0.446 | 0.043 |
| PROMIS Q38  Bloat severity | 0.137 | 0.196 | 0.226 | **0.250** | 0.277 | **0.376** | -0.165 |
| PROMIS Q39  Bloating max | 0.150 | 0.224 | 0.509* | **0.523*** | 0.281 | **0.355** | 0.132 |
| PROMIS Q40  Bloat max feel | 0.151 | 0.255 | 0.336 | **0.340** | 0.249 | **0.345** | -0.184 |
| Diarrhea | 0.169 | 0.218 | 0.031 | 0.029 | 0.266 | 0.356 | **-0.048** |
| PROMIS Q16  Frequency | 0.220 | 0.248 | 0.208 | 0.193 | 0.334 | 0.400 | **0.239** |
| Constipation | -0.144 | -0.001 | -0.233 | -0.173 | -0.276 | -0.213 | **-0.124** |
| PROMIS Q7  Frequency | -0.312 | -0.271 | -0.362 | -0.294 | -0.379 | -0.356 | **-0.065** |
| PROMIS Q8  Severity | -0.296 | -0.175 | -0.351 | -0.302 | -0.366 | -0.347 | **0.233** |
| **IBS-QOL Score** | | | | | | | |
| Overall | **0.388** | **0.427** | **0.471** | **0.475** | **0.544*** | **0.628*** | **-0.250** |

**Supporting Table 4** Correlation of the mean FAST symptoms and selected subscales and questions from the GSRS, the PROMIS GI, and the IBS-QOL in IBS-M participants

***** P-value of <0.05 is considered statistically significant.

**Bold** items denote logically related items. GSRS, gastrointestinal symptom rating scale; IBS-QOL, irritable bowel syndrome quality of life; PROMIS GI Scales, patient reported outcome measurement information system gastrointestinal scales; IBS-M, irritable bowel syndrome with mixed bo
